# A Conserved Mechanism for Sulfonucleotide Reduction

**DOI:** 10.1371/journal.pbio.0030250

**Published:** 2005-07-19

**Authors:** Kate S Carroll, Hong Gao, Huiyi Chen, C. David Stout, Julie A Leary, Carolyn R Bertozzi

**Affiliations:** **1**Department of Chemistry, University of California, Berkeley, California, United States of America,; **2**Departments of Chemistry and Molecular Cell Biology, Genome Center, University of California, Davis, California, United States of America,; **3**Department of Molecular and Cell Biology, University of California, Berkeley, California, United States of America,; **4**Department of Molecular Biology, The Scripps Research Institute, La Jolla, California, United States of America,; **5**Howard Hughes Medical Institute, University of California, Berkeley, California, United States of America; University of MichiganUnited States of America

## Abstract

Sulfonucleotide reductases are a diverse family of enzymes that catalyze the first committed step of reductive sulfur assimilation. In this reaction, activated sulfate in the context of adenosine-5′-phosphosulfate (APS) or 3′-phosphoadenosine 5′-phosphosulfate (PAPS) is converted to sulfite with reducing equivalents from thioredoxin. The sulfite generated in this reaction is utilized in bacteria and plants for the eventual production of essential biomolecules such as cysteine and coenzyme A. Humans do not possess a homologous metabolic pathway, and thus, these enzymes represent attractive targets for therapeutic intervention. Here we studied the mechanism of sulfonucleotide reduction by APS reductase from the human pathogen *Mycobacterium tuberculosis,* using a combination of mass spectrometry and biochemical approaches. The results support the hypothesis of a two-step mechanism in which the sulfonucleotide first undergoes rapid nucleophilic attack to form an enzyme-thiosulfonate (E-Cys-S-SO_3_^−^) intermediate. Sulfite is then released in a thioredoxin-dependent manner. Other sulfonucleotide reductases from structurally divergent subclasses appear to use the same mechanism, suggesting that this family of enzymes has evolved from a common ancestor.

## Abstract 2

A diverse family of enzymes that catalyze the first step in sulfur assimilation share the same mechanism.

## Introduction

Carbon, nitrogen, and sulfur undergo energy cycles in our environment that are essential to support life. While carbon and nitrogen are available primarily as gases that require fixation, sulfur occurs abundantly as inorganic sulfate. Despite this apparent advantage, a substantial energetic hurdle must still be overcome to convert the sulfur in its most oxidized form into sulfide, the oxidation state of sulfur required for the synthesis of essential biomolecules [1]. This transformation is accomplished by a group of enzymes that, together, make up the sulfate assimilation pathway [2].

**Figure 1:**
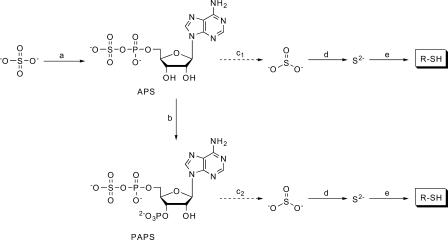
Routes of Sulfate Assimilation Inorganic sulfate is adenylated by ATP sulfurylase (reaction a) to form APS. Higher plants and the majority of sulfate reducing bacteria use APS as their source of sulfite (reactions c_1_ → d → e). In some organisms, APS kinase (reaction b) phosphorylates APS at the 3′-hydroxyl group to form PAPS for use as a sulfate donor for sulfotransferases or as a source of sulfite. The lower pathway of sulfate reduction (reactions c_2_ → d → e) is utilized by γ-proteobacteria such as *E. coli* and some fungi. Depending on the organism, APS or PAPS is reduced to sulfite by APS reductase (reaction c_1_) and PAPS reductase (reaction c_2_), respectively. Sulfite is reduced to sulfide by sulfite reductase (reaction d) and incorporated into cysteine by *O*-acetylserine-(thiol) lyase (reaction e). Important metabolites such as methionine and coenzyme A are, in turn, synthesized from cysteine.

We have studied the *Mycobacterium tuberculosis* sulfur assimilation pathway (Figure 1) as a possible venue for novel therapeutic targets [3]. In *M. tuberculosis,* ATP sulfurylase (Figure 1, reaction a) activates inorganic sulfate via adenylation. The resulting compound, adenosine 5′-phosphosulfate (APS), contains a unique high-energy anhydride bond. This vital intermediate resides at a metabolic hub within *M. tuberculosis*. In the nonreductive branch of sulfate metabolism, APS is phosphorylated a second time by APS kinase (Figure 1, reaction b) to form 3′-phosphoadenosine 5′-phosphosulfate (PAPS), the universal sulfate donor for sulfotransferases [4]. Alternatively, for the purposes of thiol or sulfide-containing metabolite production mentioned above, APS is shuttled through the reductive branch of this pathway (Figure 1, reactions c_1_, d, and e).

**Figure 2:**
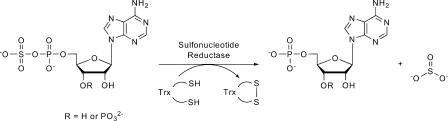
Reaction Catalyzed by Sulfonucleotide Reductases

In mycobacteria, APS reductase (Figure 1, reaction c_1_) catalyzes the first committed step in the biosynthesis of reduced sulfur compounds (Figure 2). In this reaction, APS is reduced to sulfite and adenosine 5′-phosphate (AMP) [5]. The input of electrons, or reduction potential, necessary for this reaction is provided by thioredoxin, a 12-kDa ubiquitous protein cofactor [6–8]. Thioredoxin contains a -Cys-XX-Cys- motif, and together these two cysteine residues form a redox-active disulfide bond [9]. While other interesting functions for thioredoxin have been reported, its most commonly known role is participation in thiol/disulfide exchange reactions, in which it donates electrons to proteins with concomitant oxidation of its own thiols. Notably, APS reductase has been identified in a screen for essential genes in *Mycobacterium bovis* bacillus Calmette-Guérin [10]. Furthermore, in a murine model of tuberculosis infection, APS reductase is critical for virulence (R. Senaratne, personal communication). Since humans do not possess an analogous enzyme, from a therapeutic standpoint, APS reductase represents a unique target for antibiotic therapy.

**Figure 3:**
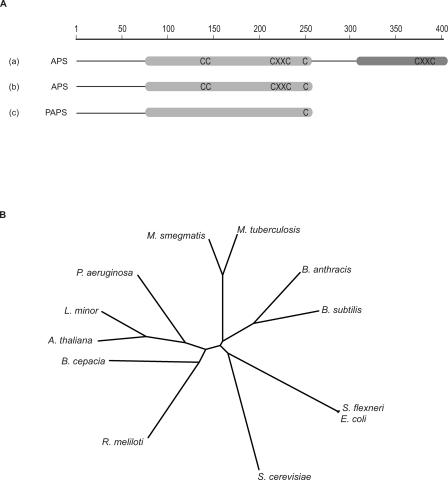
Domain Organization and Cysteine Conservation within the Sulfonucleotide Reductase Family (A) APS reductases from higher plants (group a) possess a reductase domain and a unique C-terminal domain with high homology to thioredoxin. Bacterial APS reductases (group b) lack this specialized domain, but share the cysteine motif -CC-X_∼80_-CXXC- present in the reductase domain. PAPS reductases (group c) lack this cysteine motif as well as the thioredoxin domain. All sulfonucleotide reductases have a cysteine at the end of the C terminus in the reductase domain. This residue is essential for catalysis. (B) A dendrogram illustrating the sequence homology between enzymes within the sulfonucleotide reductase family. Each of the three subclasses of sulfonucleotide reductases is clearly delineated: APS reductases from higher plants with their unique C-terminal thioredoxin domain *(A. thaliana* and *L. minor),* bacterial APS reductases *(M. tuberculosis, P. aeruginosa,* and *R. meliloti)* and PAPS-reducing organisms *(E. coli* and *S. cerevisae).* *Burkholderia cepacia, Shigella flexneri,* *B. subtilis,* and *Bacillus anthracis* were included for comparison. The sequence alignment was performed using ClustalW and the tree was constructed with the Drawtree program [53].

The requirement for reduced sulfur in the biosynthesis of cysteine, methionine, and other primary metabolites is widespread among many organisms, such as algae, plants, fungi, and a diverse array of bacteria [11–13]. However, variations within this metabolic pathway exist, and interestingly, not all organisms reduce APS. In a subtle change of substrate specificity, some organisms reduce PAPS (Figure 1, reaction c_2_) as the source of sulfite (Figure 2) [14,15]. Known collectively as sulfonucleotide reductases, this enzyme family can be subdivided into three groups based on their substrate specificity, cofactors, and domain organization (Figure 3A) [11,12,16].

The first category of reductases consists of higher plants (Figure 3A, group a), such as *Arabidopsis thaliana* and *Lemina minor* [13,17,18]. These enzymes reduce APS. The main characteristic that distinguishes this group from other sulfonucleotide reductases is the presence of a 16 kDa domain fused to the C-terminal end of the reductase, with an extremely high degree of homology to thioredoxin—in particular, the two redox-active cysteines. Notably, these enzymes also possess an iron-sulfur cluster consisting of four iron and four inorganic sulfur atoms (4Fe-4S) [18]. This cluster is ligated by the cysteines found in the sequence motif -CC-X_∼ 80_-CXXC-, although at present it is not clear whether three or all four cysteines within this motif participate in iron-sulfur cluster ligation [12,18]. On the other end of this continuum are PAPS reductases (Figure 3A, group c) found in γ-proteobacteria and in fungi, such as *Escherichia coli* and *Saccharomyces cerevisiae,* respectively [14,15]. In addition to being distinct from the first class of reductases based on substrate specificity, these enzymes lack the iron-sulfur cluster cysteine motif and are thus devoid of this cofactor.

More recently, a new subclass of sulfonucleotide reductases was discovered, designated as bacterial APS reductases (Figure 3A, group b) [11]. Representative examples from this group are found in organisms such as *Mycobacterium tuberculosis, Pseudomonas aeruginosa,* and *Rhizobium meliloti*. Interestingly, another member of this subclass, from *Bacillus subtilis,* can reduce PAPS in addition to APS [19]. These bacterial enzymes possess a higher degree of homology to plant APS reductases than to the bacterial PAPS reductases, particularly in their absolute conservation of the iron-sulfur cluster cysteine motif and 4Fe-4S cluster [12]. A dendrogram that illustrates the amino acid sequence relationship between the sulfonucleotide reductases described above is depicted in Figure 3B. Their segregation at the primary sequence level mirrors their substrate and cofactor distinctions.

Two fundamental questions remain unanswered for this family of catalysts, each subgroup with its distinct cofactors, substrate specificity and domain organization. Namely, at a molecular level, how do sulfonucleotide reductases catalyze this unique reaction and, on a broader level, do sulfonucleotide reductases utilize similar or distinct catalytic mechanisms? APS and PAPS reductases share a high degree of sequence homology and possess an absolutely conserved cysteine residue at the C terminus of the reductase domain (Figure 3A). This cysteine residue does not play a role in iron-sulfur cluster ligation, and mutation of this cysteine to serine abolishes detectable catalytic activity [12,14,18]. From these data, there is a general consensus on the essential catalytic role of this cysteine residue.

In early studies using *S. cerevisiae* and *E. coli* PAPS reductases, steady-state kinetics yielded parallel lines in Lineweaver-Burk double reciprocal plots [14,15]. Such results can be indicative of a “ping-pong” reaction mechanism. The term ping-pong is used describe a mechanism in which the enzyme first reacts with one substrate to give a covalently modified enzyme and releases one product [20,21]. The modified enzyme then reacts with a second substrate in a subsequent step. In the case of sulfonucleotide reductases, one substrate can be thought of as the sulfonucleotide and the other, thioredoxin. For simplicity, throughout the text we use the term “first step” to refer collectively to the steps that lead up to the covalently modified enzyme and the term “second step” to refer collectively to the steps subsequent to this modification.

**Figure 4:**
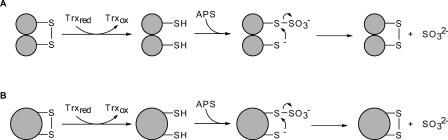
Previously Proposed Models of Sulfonucleotide Reduction (A) Prior to covalent intermediate formation, thioredoxin (Trx) reduces an intermolecular disulfide bond [14,15]. (B) Prior to covalent intermediate formation, thioredoxin (Trx) reduces an intramolecular disulfide bond [22].

Several mechanisms have been proposed for the reduction of both APS and PAPS. The fundamental difference between these mechanisms lies in the specific role of thioredoxin during the catalytic cycle. Initially, a model was hypothesized in which thioredoxin would act in the first step of the reaction catalyzed by PAPS reductase to reduce an intermolecular disulfide bond proposed to form between the two C-terminal cysteine residues of the reductase homodimer (Figure 4A) [14]. One of the liberated thiolates would subsequently execute a nucleophilic attack on the sulfur atom in PAPS, generating an enzyme-thiosulfonate intermediate, E-Cys-S-SO_3_^−^. In the second step, nucleophilic attack by the second thiolate would generate a sulfite product with concomitant reoxidation of the intermolecular disulfide bond.

In a more recent study, it was proposed that APS reductase from *P. aeruginosa,* and likely all bacterial APS reductases, employ a slight variant of the PAPS reductase catalytic strategy (Figure 4B) [22]. In this work, Kim and colleagues suggested that thioredoxin reduces an intramolecular disulfide bond proposed to form between the C-terminal thiol in the reductase domain and the second cysteine in the -CC- sequence motif. The remainder of the reaction would proceed as previously put forward for PAPS reductase; the partner thiolate in the reduced disulfide bond would attack the thiosulfonate intermediate to regenerate the intramolecular disulfide and yield sulfite product.

An alternative mechanism has been suggested for the reduction of APS in higher plants such as *A. thaliana* and related organisms that possess the C-terminal thioredoxin domain [23]. In this model, APS binds to the reductase in the first step and forms an enzyme-thiosulfonate intermediate via the essential C-terminal cysteine. In a subsequent step, the authors propose that the thioredoxin domain would be responsible for the release of bound sulfite. In a series of experiments using only the N-terminal APS reductase domain of the enzyme, Weber and colleagues convincingly established the presence of a covalent sulfite adduct at the C-terminal cysteine in the reductase domain [23]. Demonstration of a specific requirement for thioredoxin in product release remained more elusive, as the N-terminally truncated enzyme was only 3-fold more active in the presence of recombinant thioredoxin *m*, as compared to dithiothreitol (DTT) alone. A second thioredoxin, thioredoxin *f*, provided no catalytic advantage over DTT. Furthermore, as this small group of reductases from higher plants possessed different domain organization from the bulk of the enzyme family, it was unclear if the results from this work could be more generally applied.

Herein, we describe experiments that draw upon a combination of approaches—biophysical analysis, site-directed mutagenesis, kinetics, cysteine labeling, and direct analysis of enzyme intermediates by Fourier transform ion-cyclotron resonance (FT-ICR) mass spectrometry—to address the question of mechanism in sulfonucleotide reductases. Our results demonstrate that *M. tuberculosis* APS reductase catalyzes the reduction of APS in a two-step process: In the first step, APS undergoes rapid nucleophilic attack to form a unique enzyme-thiosulfonate intermediate. In the second step, thioredoxin mediates sulfite release.

We also investigated the mechanisms of other bacterial APS reductases, including that from *P. aeruginosa*. In contrast to previous suggestions, all bacterial APS reductases we studied utilize the same general mechanism as we found for *M. tuberculosis* APS reductase. Additionally, we have reexamined the mechanism proposed for PAPS reduction by *E. coli* PAPS reductase. Like the APS reductases, *E. coli* PAPS reductase forms an enzyme-thiosulfonate intermediate and employs the same overall catalytic strategy as all other sulfonucleotide reductases investigated in this study. Taken together, these data suggest that the family of sulfonucleotide reductases share the same general catalytic strategy and provide functional evidence for evolution from a common ancestor.

## Results

The first mechanism proposed for sulfonucleotide reduction was generated from studies with *S. cerevisiae* and *E. coli* PAPS reductases [14,15]. Both enzymes were isolated as homodimers and possessed a single cysteine at their C termini. It was proposed that reduction of a dynamic intermolecular disulfide bond formed between the two C-terminal cysteine residues initiated the catalytic cycle (Figure 4A). One of the liberated thiolates would attack the substrate sulfur atom to form a thiosulfonate intermediate. Subsequent attack by the second cysteine upon this intermediate would regenerate the enzyme disulfide and produce sulfite. There are four clear predictions from this model: (1) Sulfonucleotide reductases should be homodimers; (2) In the first step of the reaction, thioredoxin should reduce an intermolecular disulfide bond between the catalytic cysteines; (3) A thiosulfonate enzyme intermediate should be formed; and (4) The second thiolate should be essential for regeneration of the proposed disulfide bond and the production of sulfite. As *M. tuberculosis* APS reductase is a compelling target for drug development, we wanted to test the various features of this mechanism in order to better inform our efforts at rational drug design.

### *M. tuberculosis* APS Reductase Is a Monomer

**Figure 5:**
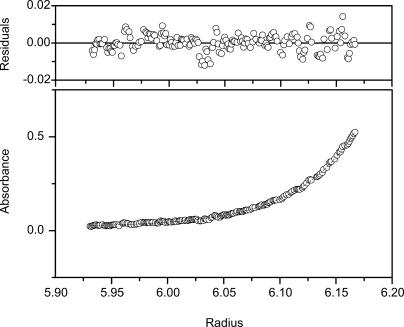
M. tuberculosis APS Reductase Is a Monomer Equilibrium sedimentation experiments of *M. tuberculosis* APS reductase were performed using a Beckman Optima XL-I centrifuge at 4 °C. The concentration of APS reductase shown in this figure was 10 μM. However, as described in Materials and Methods, multiple concentrations of enzyme were analyzed, and in all cases we observed that the enzyme was a monomer. Fitting the data to a model of a single ideal species yielded a molecular mass of approximately 28.5 kDa.

We set out to confirm that *M. tuberculosis* APS reductase was a dimer, but to our surprise, size exclusion chromatography of *M. tuberculosis* APS reductase indicated that this enzyme was a monomer (Figure S1A). Under the same conditions, *E. coli* PAPS reductase eluted exclusively as the homodimer, as reported in previous work (Figure S1A) [14]. To rule out the possibility of anomalous migration on the gel filtration column, we used analytical ultracentrifugation to determine the molecular weight of *M. tuberculosis* APS reductase. Figure 5 depicts representative data from these experiments from a sample containing 10 μM enzyme. Analysis of these data yielded an estimated molecular weight for *M. tuberculosis* APS reductase of 28,513 Daltons (Da). This value is in excellent agreement with the expected molecular weight of the holoenzyme, 28,705.88 Da. As an independent confirmation of these results, an electrospray ionization (ESI) mass spectrum was acquired for APS reductase. The enzyme was sprayed in ammonium acetate buffer, a mild condition that enables the analysis of the folded protein with retention of noncovalently associated cofactors such as an iron-sulfur cluster (referred to as “native mass analysis” throughout the text) [24,25]. The mass spectrum of APS reductase showed a charge distribution of 9+, 10+, and 11+ with a mass of 28,706.0 ± 0.06 Da, corresponding to the monomeric protein (Figure S1B).

To ensure that the histidine (His) tag was not disrupting oligomerization, untagged *M. tuberculosis* APS reductase was expressed and purified. This enzyme migrated with the same retention volume on the size exclusion column as His-tagged APS reductase (Figure S2A). To test for oligomerization in the presence of substrate, AMP, or thioredoxin, *M. tuberculosis* APS reductase was analyzed by successive runs on a gel filtration column whose buffer contained a saturating amount of the ligand or protein tested. There was no evidence of dimerization under any condition (unpublished data). APS reductase activity for the untagged and His-tagged protein was also assayed across the protein elution profile of the size exclusion column. In both cases, only a single peak of activity was observed; the elution volume of this activity corresponded to the monomeric molecular weight (Figure S2). The specific activity of His-tagged and untagged *M. tuberculosis* APS reductase using *E. coli* thioredoxin were 5.1 and 4.6 μmol min^−1^ mg protein^−1^, respectively. These values are within range of those previously reported for APS reductases from other organisms such as *P. aeruginosa* (5.8 μmol min^−1^ mg protein^−1^), *R. meliloti* (7 nmol min^−1^ mg protein^−1^), *B. subtilis* (0.28 μmol min^−1^ mg protein^−1^), and *A. thaliana* (30 μmol min^−1^ mg protein^−1^) [11,16,19,23]. Taken together, these experiments demonstrate that the active form of *M. tuberculosis* APS reductase is monomeric. Furthermore, this finding was not confined to a single species as other bacterial APS reductases such as those from *Mycobacterium smegmatis* and *R. meliloti* were also isolated as monomers (unpublished data). These data, in addition to the continuing controversy regarding differing mechanisms proposed for this family of enzymes, prompted us to investigate the mechanism of *M. tuberculosis* and other sulfonucleotide reductases in more detail.

### *M. tuberculosis* APS Reductase Does Not Contain an Intramolecular Disulfide Bond

One possible explanation for these data was that monomeric sulfonucleotide reductases might possess an intramolecular disulfide bond instead of an intermolecular disulfide bond. Indeed, Kim and colleagues have recently made this proposal to describe the catalytic behavior of *P. aeruginosa* and bacterial APS reductases in general [22]. In this slight variation of the original model, thioredoxin would reduce an intramolecular disulfide bond in the first step with all subsequent steps remaining the same (Figure 4B). To investigate whether an intramolecular disulfide bond existed in *M. tuberculosis* APS reductase, cysteine-labeling experiments were carried out using 4-vinylpyridine (VP), a small and highly specific reagent used to covalently modify free thiols. After cysteine-labeling and buffer exchange to remove unreacted reagent, the number of covalently modified cysteines was determined by denatured mass analysis (treatment of enzyme with 80% acetonitrile and 1% formic acid). First, we confirmed that all six cysteines could be labeled in the unfolded enzyme. When the enzyme was denatured with guanidinium hydrochloride in the presence of the reducing agent tris-(2-carboxyethyl)phosphine (TCEP), reaction with a 2-fold excess of VP over the total concentration of cysteine residues resulted in five labeled cysteines. Reaction with a 10-fold excess of reagent successfully labeled all six cysteines (unpublished data). Next, we probed for cysteine modifications in the folded protein. Incubation of the native enzyme with a 10-fold excess of VP under reducing conditions yielded a molecular weight shift that corresponded to the addition of only two vinyl pyridine labels (Table 1). No additional labels were observed upon increasing the amount of VP to a 100-fold excess over total cysteine concentration in the presence of either TCEP or DTT. Moreover, no change in labeling was observed when reductant was omitted from labeling reactions (unpublished data). These results suggested that only two reactive thiols were accessible within the folded *M. tuberculosis* APS reductase protein.

**Table 1:**
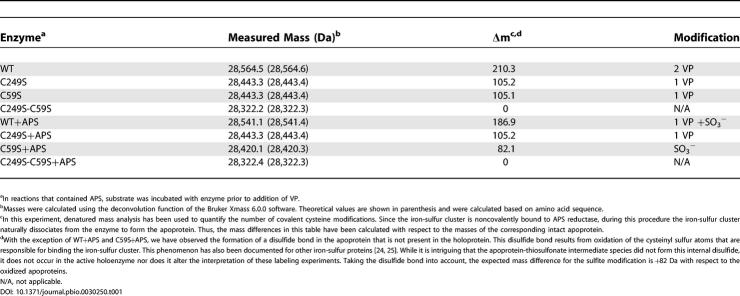
Mass Measurements of Wild-Type and Mutant *M. tuberculosis* APS Reductase Labeled with VP

From sequence gazing, we speculated that the two most likely candidates responsible for the modifications were cysteine 59 (Cys59), a nonconserved cysteine present in the N-terminal portion of the enzyme, and cysteine 249 (Cys249), the most C-terminal cysteine and putative catalytic nucleophile. To test whether these were indeed the cysteines accessible to VP labeling, the same labeling experiments were carried out with two cysteine-to-serine mutant enzymes. Since VP does not label serine residues, mutation of Cys59 or Cys249 to serine would result in the loss of a single label if they represented the labeled species in the wild-type enzyme. As predicted, each mutant resulted in the loss of precisely one VP label (Table 1). Furthermore, no labels were obtained with the double mutant Cys59Ser-Cys249Ser (Table 1). These experiments confirmed the identity of the VP-modified residues in the wild-type enzyme as Cys59 and Cys249.

**Figure 6:**
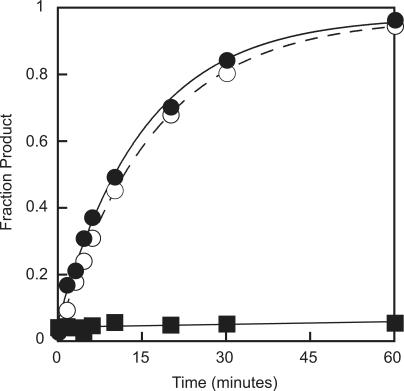
Cysteine 59 Is Not Required for *M. tuberculosis* APS Reductase Activity The activity of wild-type (closed circles), Cys59Ser (open circles) and Cys249Ser (closed squares) APS reductase was determined as described in Materials and Methods. The concentration of sulfonucleotide reductase in this experiment was 10 nM and the concentration of APS was 20 μM.

The thiol labeling experiments demonstrated that *M. tuberculosis* APS reductase contains two accessible cysteine side chains that could, in theory, form an intramolecular disulfide bond. However, when the molecular weight of native *M. tuberculosis* APS reductase holoenzyme was evaluated by native mass spectrometry, we found no evidence for the presence of a disulfide bond, as the measured mass, in the absence or presence of reductant, was 28,706.0 ± 0.06 Da. This mass is in excellent agreement with the theoretical value of 28,705.88 Da, the predicted molecular weight for this enzyme without an intramolecular disulfide bond. Furthermore, when we assayed the mutant enzymes for catalytic activity, we found that Cys59 was entirely dispensable for catalysis (Figure 6), which strongly suggests that no catalytically essential disulfide bond with this residue was formed. In contrast, the Cys249Ser mutation resulted in an inactive enzyme, as anticipated from previous work demonstrating the essential nature of this residue (Figure 6). Taken together, these experiments demonstrate that intramolecular disulfide bond formation does not play a role in substrate reduction by *M. tuberculosis* APS reductase.

### A Stable Thiosulfonate Intermediate Is Formed in the Absence of Thioredoxin

Prior work with sulfonucleotide reductases from other organisms, as well as the data described above, had shown that the C-terminal cysteine residue (Cys249) in the reductase domain of *M. tuberculosis* APS reductase was essential for activity [14,23]. However, we have also shown that this cysteine is not involved in the formation of a disulfide bond that would require reduction by thioredoxin prior to substrate attack. We therefore considered the possibility that this cysteine would act directly as a nucleophile in attacking APS, without prior reduction by thioredoxin, to yield an enzyme-thiosulfonate intermediate. While predicted by the original model proposed for PAPS reduction, the existence of such an intermediate has been under debate for some time in the sulfonucleotide reductase literature [15,22,23,26]. Incubation of APS with a truncated version of *A. thaliana* APS reductase under physiological conditions yielded a sulfite bound to the C-terminal cysteine within the reductase domain [23]. Despite these intriguing data, no definitive evidence for such an intermediate has been presented for any bacterial APS or PAPS reductase.

**Figure 7:**
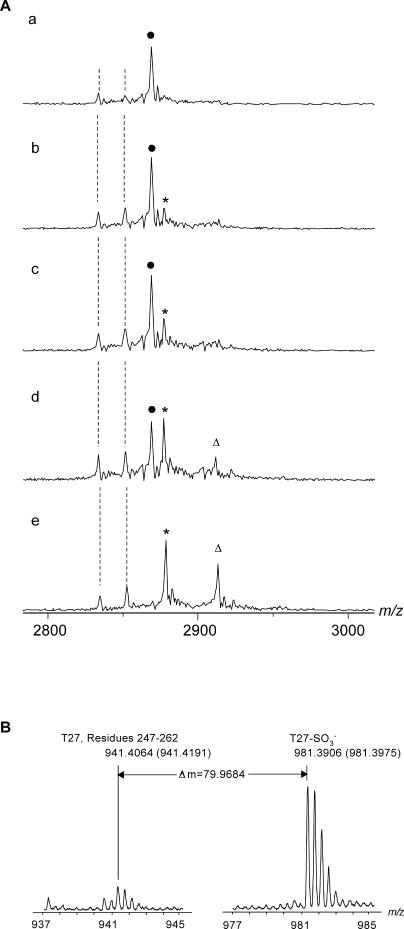
M. tuberculosis APS Forms a Thiosulfonate-Enzyme Intermediate in the Absence Thioredoxin (A) ESI mass spectra in low resolution showing the titration of APS reductase with APS in 50 mM NH_4_OAc (pH 7.5). In tracings labeled a to e, the concentration of APS was 0, 1, 2, 4, and 10 μM, respectively. The concentration of APS reductase is 15 μM in all cases. Peaks indicate ions corresponding to the 10+ charge states of the enzyme (E, solid circles), the covalent intermediate (E-SO_3_^−^, asterisks), and the noncovalent complex between the intermediate and AMP (E-SO_3_^−^ • AMP, triangles). The dashed lines highlight the ions of APS reductase that lack a mature iron-sulfur cofactor. The concentrations of APS reductase reported in this figure were measured prior to mass analysis. A small fraction of protein loss occurs during the mass analysis, and thus the reported concentrations of protein should be treated as an upper limit. (B) ESI mass spectra of trypsin-digested APS reductase incubated with a 10-M excess of APS. The C-terminal peptide (T27, residues 247–262) containing Cys249 showed a +80 Da shift (right tracing) corresponding to the molecular weight of a sulfite compared to the unmodified peptide (left tracing). The measured *m/z* values of the peptide with and without bound sulfite are indicated in the spectra. Shown in parenthesis are the theoretical values.

With *M. tuberculosis* APS reductase, we hypothesized that the intermediate could be accumulated simply by omitting thioredoxin from the reaction. In order to test this model, we used mass spectrometry to probe for modifications to the enzyme during the reaction. In these experiments, APS was incubated with enzyme in ammonium acetate buffer; the mixture was sprayed directly for native mass spectrometry analysis. As reported above, the mass spectrum of APS reductase in the absence of substrate is characterized by a major series of ions with a molecular weight corresponding to the holoenzyme (Figure 7A, solid circles). Coincubation of APS reductase and APS resulted in the formation of a new series of ions with a molecular weight approximately 80 Da higher than the holoenzyme (Figure 7A, asterisks). Moreover, the signal of these ions intensified in response to increasing APS concentration. High-resolution FT-ICR mass analysis of the new species confirmed a mass shift of +80.04 ± 0.07 Da relative to the holoenzyme, consistent with the molecular weight expected for a covalently bound sulfite. Finally, proteolytic digest of the intermediate followed by mass analysis of the resulting peptides confirmed the exact mass difference of +80 Da relative to the unmodified peptide (Figure 7B). Thus, the simplest interpretation of these data is that incubation of substrate with enzyme produces a covalently bound sulfite modified intermediate.

To support the mass spectrometry findings, biochemical assays were also performed. Incubation of [^35^S]-labeled APS together with enzyme, and subsequent analysis of the reaction by nonreducing SDS-PAGE, produced a single radioactive band—indicating the transfer of the ^35^S label from the substrate to the enzyme—at the expected molecular weight for APS reductase (Figure S3, lane 1). This radiolabel could be eliminated in the unfolded enzyme by heat in combination with β-mercaptoethanol (unpublished data). Likewise, preincubation of enzyme with saturating amounts of nonradioactive substrate prior to addition of [^35^S]APS, blocked radiolabeling (Figure S3, lane 2). Taken together, these data demonstrate the formation of a stable thiosulfonate enzyme-intermediate in *M. tuberculosis* APS reductase.

In the mass spectrometry analysis of APS reductase incubated with APS, we also detected a third series of ions with a calculated mass corresponding to the holoenzyme plus 427 Da (Figure 7A, triangles). The additional 427 Da could represent the intact holoenzyme bound to unreacted substrate, APS. Alternatively, this species could represent the enzyme-thiosulfonate intermediate bound to the second product, AMP. To distinguish between these two possibilities, we carried out solution kinetic experiments using conditions identical to those used for mass spectrometry to measure the rate at which substrate was converted to intermediate. By the first measurable time point (15 s), all substrate had been consumed, suggesting that the +427 Da ion was not due to binding of unreacted substrate (unpublished data). Therefore, the simplest interpretation of these data is that the third ion represents APS reductase bound covalently to sulfite and noncovalently to the product, AMP. Finally, in this experiment we also observed two minor series of ions whose molecular weights were less than the holoenzyme (Figure 7A, dashed lines). The mass of these ions was 28,352.4 Da and 26,529.3 Da. It has been well established that iron-sulfur clusters are vulnerable to oxidation and dissociation from their protein scaffolds [18,19,25,27]. For *M. tuberculosis* APS reductase, the theoretical mass for the apoenzyme (enzyme without iron-sulfur cluster) is 28,352.2 Da and for the 2Fe-2S intermediate it is 28,529.1 Da. These values are within 0.2 Da of the measured masses of the ions noted above. Therefore, a likely possibility is that these ions represent different stages in cluster decomposition of APS reductase. In contrast to the holoenzyme, these lower molecular weight ions did not exhibit any mass shift upon the addition of APS.

### The Thiosulfonate Intermediate Is Bound to the C-Terminal Cysteine

**Figure 8:**
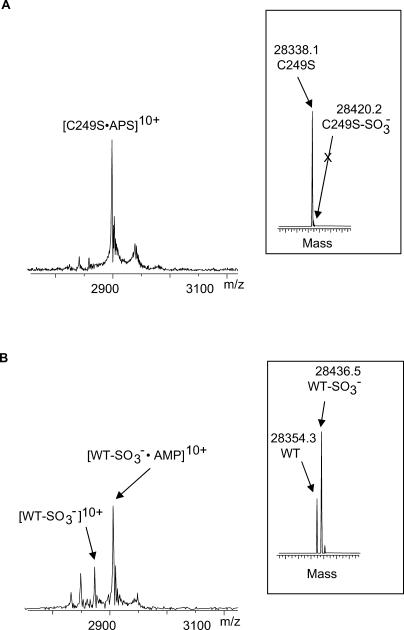
M. tuberculosis APS Reductase Cys249Ser Mutant Enzyme Binds APS, but Does Not Form a Covalent Intermediate ESI mass spectra of 20 μM Cys249Ser APS reductase with 20 μM APS (A) and wild-type enzyme (B) with 5 μM APS in 50 mM NH_4_OAc (pH 7.5). Insets: The corresponding deconvoluted spectra acquired in 80% acetonitrile showing the presence and absence of a covalent modification to the mutant and wild-type enzymes, respectively.

To test whether the C-terminal cysteine, Cys249, was essential for the formation of the enzyme-sulfite intermediate, we carried out experiments analogous to those above with the Cys249Ser mutant. If Cys249 is the nucleophile for attack on APS, the Cys249Ser mutant should not form the enzyme-sulfite intermediate. In these experiments, APS was incubated with the Cys249Ser mutant APS reductase. When this reaction was analyzed by native mass spectrometry, the mutant protein exhibited a mass shift of +427 Da, consistent with the molecular weight of APS (Figure 8A). However, despite the ability of the mutant to bind APS, this variant did not form the thiosulfonate enzyme-intermediate. In separate experiments, after incubation with substrate, the Cys249Ser mutant enzyme was treated with 80% acetonitrile and 1% formic acid to unfold the protein and protonate the side chains for denatured mass spectrometry analysis. This treatment resulted in release of the noncovalently bound APS from the mutant enzyme, and only the intact apoprotein was observed (Figure 8A, inset). In contrast, native mass analysis of the reaction between APS and wild-type APS reductase yielded the expected thiosulfonate-enzyme intermediate. Subsequent analysis of this intermediate under denaturing conditions demonstrated that the sulfite modification persisted, as expected for a covalently bound intermediate (Figure 8B; Figure 8B, inset).

As an independent confirmation of these results and a more direct demonstration that the sulfite was attached specifically to Cys249, cysteine-labeling experiments were carried out with wild-type APS reductase and the two mutants, Cys59Ser and Cys249Ser, using VP as a probe of cysteine reactivity after incubation with APS. If the sulfite intermediate were bound to Cys249, this residue would be unreactive toward VP labeling. Therefore, in the presence of substrate, both wild-type and Cys59Ser enzymes should lose a single VP label with a concomitant gain of a sulfite, while Cys249Ser should only lose a single VP label. When the products of these reactions were analyzed by denatured mass spectrometry, each of these expectations was confirmed (Table 1). Finally, when Cys249Ser was incubated with [^35^S]-labeled APS and analyzed by nonreducing SDS-PAGE, no radiolabeled protein resulted (Figure S3, lane 4), in contrast to Cys59Ser (Figure S3, lane 7) and wild-type enzyme (Figure S3, lane 1). These data demonstrate that the C-terminal cysteine residue (Cys249) is essential for intermediate formation, and confirm that the sulfite was bound to this catalytic cysteine.

### Thioredoxin Is Not Required for Intermediate Formation but for Product Formation

The previous experiments suggested that incubation of substrate together with enzyme is sufficient to form the thiosulfonate enzyme-intermediate. The simplest interpretation of these data is that formation of the covalent intermediate occurs prior to a step that requires thioredoxin. Two predictions from this model are that (1) one equivalent of covalent intermediate per equivalent of active enzyme is formed in the absence of thioredoxin, and (2) the addition of thioredoxin will lead to product formation and turnover of the enzyme.

**Figure 9:**
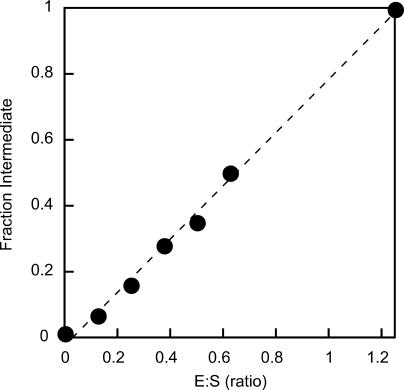
Covalent Enzyme-Intermediate Formation Is Stoichiometric with APS Reductase The fraction of APS converted to thiosulfonate enzyme-intermediate was monitored as a function of APS reductase concentration as described in the Materials and Methods. The concentration of APS in each reaction was 0.5 μM. Enzyme concentration was varied between 0 and 1.25 μM. The dashed line is a theoretical fit of the fraction intermediate formed and its dependence upon enzyme concentration to a linear equation (*R* ^2^ ≥ 0.98).

To test the first prediction, we quantified formation of thiosulfonate enzyme-intermediate as a function of APS reductase concentration. The data from this experiment are shown in Figure 9; for ease of analysis we have plotted the ratio of enzyme to substrate concentration (E:S) as the x-coordinate. The fraction of intermediate formed increased linearly with enzyme concentration. At an equimolar concentration of enzyme and substrate, 80% of the substrate would be converted into the covalent intermediate. Depletion of substrate at an exact one-to-one ratio of enzyme to substrate would require that our enzyme preparation contain 100% active molecules. Thus, taking into account that oxidation of the iron-sulfur cluster results in inactive enzyme (see Figure 7) [18,19], these experiments show that the fraction of covalent intermediate formed relative to enzyme concentration is robust and relevant to the mechanism.

**Figure 10:**
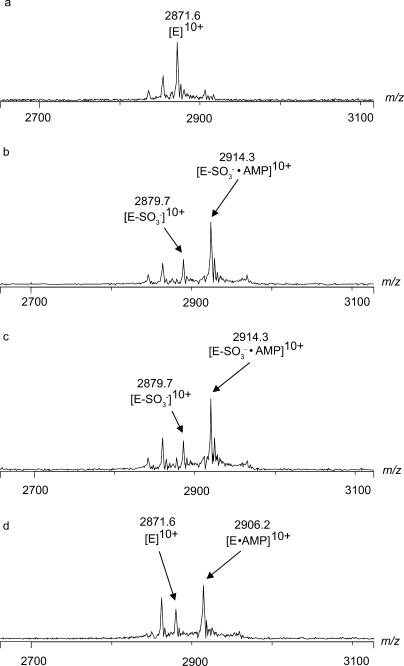
Thioredoxin Reduces the Thiosulfonate Enzyme-Intermediate ESI mass spectra, in low resolution, of 10 μM APS reductase (A); 2.5 μM APS and 10 μM APS reductase (B); 2.5 μM APS, 3.7 μM oxidized thioredoxin, and 10 μM APS reductase (C); and 2.5 μM APS, 3.7 μM thioredoxin reduced with 33 μM DTT, and 10 μM APS reductase (D). The ions corresponding to the 10+ charge state of the enzyme-associated species are labeled.

The second prediction of our model is that thioredoxin will reduce the enzyme-thiosulfonate intermediate to form product. This hypothesis was first tested using native mass analysis. In the absence of substrate, the spectrum of APS reductase was characterized by three series of ions; the major series of ions corresponded to the holoenzyme (Figure 10). Upon supplementation with substrate, the enzyme-thiosulfonate intermediate was formed. Addition of reduced, but not oxidized, thioredoxin resulted in the release of the intermediate. Control experiments demonstrated that release of sulfite was not due to residual DTT in the reduced thioredoxin sample (unpublished data).

**Figure 11:**
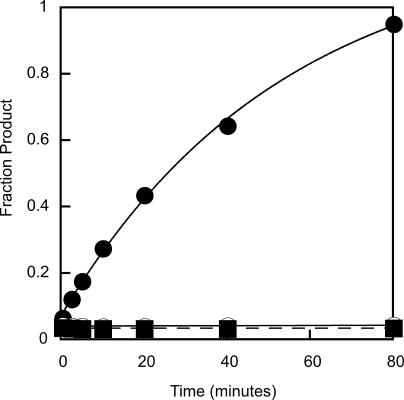
Sulfite Release Is Thioredoxin-Dependent Comparison of substrate turnover for *M. tuberculosis* APS reductase supplemented with 10 μM thioredoxin (closed circles), with 10 mM DTT (open circles), or in the absence of all reductants (closed squares) as described in the Materials and Methods. The concentration of APS reductase in this experiment was 5 nM, and of APS was 20 μM.

Solution kinetics and gel-labeling experiments were also performed to verify these mass spectrometry data. Addition of thioredoxin to a reaction mixture containing APS reductase and APS enabled multiple turnover of substrate (Figure 11). Sulfite was not released by the addition of high concentrations of DTT in place of thioredoxin as the reductant. Additionally, a panel of small-molecule reductants with varying reduction potentials was screened for their ability to release sulfite from the intermediate. In each case, APS reduction was below our detection limit over the time scale of the experiment (≤ 10^−5^ min^−1^, unpublished data). Finally, gel-labeling experiments demonstrated that incubation of [^35^S]-SO_3_^2−^ radiolabeled reductase together with thioredoxin resulted in release of the radiolabel, as expected (Figure S3, lanes 3 and 9). These experiments demonstrated that efficient turnover of APS specifically requires thioredoxin.

### Sulfonucleotide Reduction Revisited

Given the results for *M. tuberculosis* APS reductase and their consistency with the mode of action proposed for *A. thaliana* APS reductase, we wondered whether all sulfonucleotide reductases utilized the same overall catalytic strategy. To further test this hypothesis, we chose to investigate *E. coli* PAPS reductase and *P. aeruginosa* APS reductase, which have been proposed to act via the mechanisms presented in Figure 4A and 4B, respectively.

With these two enzymes, we performed mass spectrometry analysis and biochemical characterization similar to that described for *M. tuberculosis* APS reductase. First, we probed for thiosulfonate formation. Like *M. tuberculosis* APS reductase, both *E. coli* PAPS reductase and *P. aeruginosa* APS reductase formed the enzyme-thiosulfonate intermediate without prior reduction by thioredoxin (Table 2). As expected, mutation of the catalytic cysteine in each enzyme to serine prevented intermediate formation (Table 2). Also consistent with *M. tuberculosis* APS reductase, the covalently bound sulfite was released upon addition of reduced thioredoxin and not by DTT (Table 2). These conclusions were further corroborated as described above for *M. tuberculosis* APS reductase using data obtained from gel-labeling and biochemical assays (Figures S4 and S5). Finally, two additional bacterial APS reductases from *R. meliloti* and *M. smegmatis* were analyzed in similar biochemical experiments (unpublished data). These studies reiterated the findings observed with all other sulfonucleotide reductases analyzed in this study—the reaction proceeded via the covalent sulfite-bound intermediate; sulfite production was dependent upon reduction of this intermediate by thioredoxin.

**Table 2:**
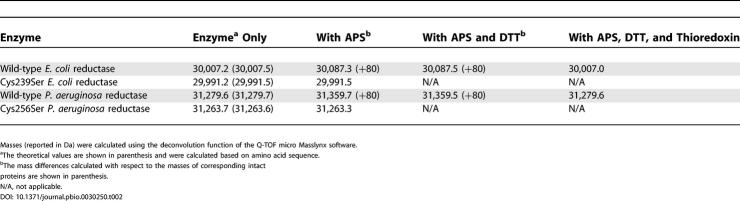
Mass Measurements of Wild-Type and Mutant *E. coli* and *P. aeruginosa* Reductases Reacted with Substrate, in the Absence or Presence of Thioredoxin

To complete our analysis, we carried out cysteine-labeling with *E. coli* PAPS reductase and *P. aeruginosa* APS reductase using VP. Since *E. coli* PAPS reductase contains only one cysteine residue in its amino acid sequence, incorporation of one VP label was clearly predicted. A single label was obtained and, as described for *M. tuberculosis* APS reductase, this labeling could be blocked by preincubation of substrate and enzyme prior to the addition of VP (Table 3). The primary amino acid sequence of *P. aeruginosa* APS reductase contains five cysteine residues, four from the -CC-X_∼ 80_-CXXC- sequence motif and one from the C-terminal cysteine nucleophile. When *P. aeruginosa* APS reductase was incubated with VP and the reaction products were analyzed by mass spectrometry, as in the labeling studies with *E. coli* and *M. tuberculosis* reductases, only a single VP label was observed (Table 3). To ensure that this pattern of labeling was not an artifact specific to VP modification, we repeated the experiments with iodoacetamide, another cysteine-modifying reagent. As in labeling studies with VP, a single label was obtained with iodoacetamide under reducing or oxidizing conditions (unpublished data). Site-directed mutagenesis as well as preincubation of the enzymes with substrate prior to VP addition positively identified the labeled residue as the C-terminal cysteine (Tables 2 and 3). These experiments provide no evidence to support the existence of a catalytic disulfide bond in either *E. coli* PAPS reductase or *P. aeruginosa* APS reductase. Moreover, based on the predictions made by the models presented in Figure 4A and 4B, reoxidation of such a disulfide bond would preclude the isolation of a stable enzyme-thiosulfonate intermediate and directly contradict the observed requirement for thioredoxin in sulfite release. These results demonstrate in *E. coli* PAPS and *P. aeruginosa* APS reductases that (1) incubation of sulfonucleotide reductase together with substrate yields a covalent thiosulfonate-enzyme intermediate; (2) intermediate formation is not dependent upon prior reduction of the sulfonucleotide reductase by thioredoxin; and (3) thioredoxin is essential for reduction of the thiosulfonate intermediate.

**Table 3:**
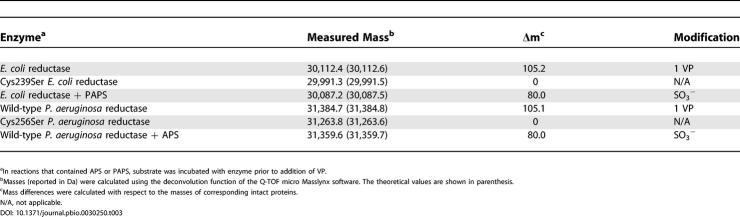
Mass Measurements of Wild-Type and Mutant *E. coli* PAPS and *P. aeruginosa* APS Reductases Labeled with VP

## Discussion

Over the last decade, a large body of work has contributed substantially to our understanding of the unique family of sulfonucleotide reductase enzymes. The authors of these studies have proposed models for sulfonucleotide reduction that have predictive power. We have anchored our studies by systematically testing some of the central predictions from the leading models previously proposed for sulfonucleotide reduction. In this study, we have used new experimental approaches to investigate this reaction from a different perspective—namely, through the use of high-resolution FT-ICR mass spectrometry in conjunction with cysteine-labeling, solution-based kinetic analysis, and other biochemical approaches. Our results strongly suggest that a general catalytic strategy is shared by all sulfonucleotide reductases and provide the first functional evidence for evolution from a common ancestor.

### A Universal Mechanism for Sulfonucleotide Reduction

**Figure 12:**
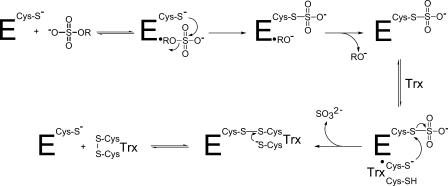
Mechanism of Sulfonucleotide Reduction

The data presented in this paper demonstrate that each of the bacterial sulfonucleotide reductases operates via a two-step mechanism, as originally proposed by Weber and coworkers to describe APS reduction in higher plants [23]. In Figure 12, we take the original proposal by Weber and coworkers a step further and provide a detailed picture of the molecular events that might take place during sulfonucleotide reduction. During the first step, an absolutely conserved cysteine residue carries out nucleophilic attack on the sulfonucleotide sulfate group to yield an enzyme-thiosulfonate intermediate. The stable formation of this intermediate has been demonstrated for each enzyme investigated here. In all cases, addition of substrate to enzyme with subsequent analysis of the reaction mixture by mass spectrometry yielded holoenzyme with a molecular weight shift that corresponds the covalent addition of a sulfite group (see Figures 7 and 8; Table 2). Additionally, gel-labeling experiments have demonstrated the requisite transfer of radiolabel from [^35^S]-substrate to enzyme (Figures S3 and S4). We have also shown through site-directed mutagenesis and cysteine-labeling studies that the conserved nucleophile is not only essential for intermediate formation, but is also the site of sulfite attachment (see Figures 8, S3, and S4; Tables 2 and 3). Pre-steady state kinetic analysis shows that sulfonucleotide reductases are blocked after covalent intermediate formation in the absence of thioredoxin and cannot perform multiple rounds of substrate turnover (see Figures 9 and S5). Finally, native mass analysis of *M. tuberculosis* APS reductase demonstrates that there are no disulfide bonds in the holoenzyme or in the thiosulfonate enzyme-intermediate; cysteine-labeling studies further support this conclusion for each of the sulfonucleotide reductases analyzed in this work (see Tables 1 and 3). Taken together, these experiments demonstrate that thioredoxin is not required for sulfonucleotide binding and enzyme-thiosulfonate intermediate formation.

In the second half of the reaction, we demonstrated that thioredoxin is required for product formation. Mass spectrometry and gel-labeling experiments clearly show the resolution of thiosulfonate enzyme-intermediate with concomitant regeneration of free sulfonucleotide reductase when thioredoxin is included in the reaction mixture (see Figures 10, 11, and S5; Table 2). In addition, these studies demonstrate that reduction of the thiosulfonate bond requires thioredoxin; DTT by itself does not efficiently support the reduction of the intermediate (see Figures 10, 11, and S5; Table 2). In the final steps of the reaction, we propose that the more reactive N-terminal thioredoxin cysteine would carry out nucleophilic attack upon the sulfonucleotide reductase catalytic cysteine (Figure 12) [9]. This reaction would result in the formation of a mixed disulfide between thioredoxin and the sulfonucleotide reductase and, concomitantly, release sulfite. Subsequent thiol/disulfide exchange with the second thioredoxin thiolate would yield oxidized thioredoxin and regenerate reduced sulfonucleotide reductase. The proposed covalent protein-protein intermediate is reminiscent of those predicted during the thioredoxin-mediated reduction of protein disulfides [9]. However, the subsequent chemical step is very rapid and we find no evidence for accumulation of this species. This result is not unexpected, because of the intramolecular nature of this reaction; we are currently using different experimental approaches to test this aspect of the proposed model in greater experimental detail.

A commonly employed assay to measure sulfonucleotide reductase includes the addition of 20–40 mM nonradioactive sulfite during the reaction or at its conclusion [13,28]. In their work with *A. thaliana* APS reductase, Weber et al. [23] demonstrated that the sulfite bound to the C-terminal cysteine is released upon exposure to millimolar concentrations of sulfite, a natural reductant. We have reiterated these findings in our own studies (unpublished data). Thus, the inclusion of sulfite in the assay of sulfonucleotide reductase activity results in thioredoxin-independent reduction of a thiosulfonate enzyme-intermediate to produce sulfite. It is likely that this phenomenon has hampered prior discovery of the covalent intermediate in bacterial sulfonucleotide reductases, and can account for the modest activity observed in the absence of thioredoxin reported by previous studies [11,12,14].

### A Novel Enzyme-Thiosulfonate Intermediate

The thiosulfonate species (E-Cys-S-SO_3_^−^) formed during sulfonucleotide reduction is novel among the diverse array of characterized covalent enzyme intermediates. The most closely related covalent enzyme-intermediate is the persulfide (E-Cys-S-S^−^) found in the family of NifS-like enzymes that includes NifS from *Azotobacter vinelandii* and *E. coli* IscS [29,30]. These catalysts have cysteine desulfurase activity that is utilized to mobilize sulfur from L-cysteine [29–31]. The sulfur is bound to a conserved active site cysteine residue from which is it subsequently transferred to form a persulfide group on a variety of other targets, such as the scaffold proteins NifU and IscU, for use in the assembly of iron-sulfur clusters [32]. In addition to cluster formation, NifS-like enzymes are also required for other biological processes such as the biosynthesis of thiamin, biotin, and molybdenum cofactors [32,33]. Another catalyst that has been demonstrated to form a persulfide intermediate is the sulfur transferase rhodanese. Like NifS-like proteins, this enzyme cycles between a sulfur-free and a stable persulfide-containing form [34]. In vitro, it has been demonstrated that rhodanese can act as a sulfur insertase and regenerate iron-sulfur clusters in proteins [35]. However, the in vivo role of rhodanese has not been well established. Notably, the structure of sulfur-free rhodanese and its persulfide-containing form have been solved for a number of organisms [34,36]. A comparison of the two enzyme species indicates a significant conformational rearrangement in the vicinity of the catalytic cysteine upon persulfide formation. By analogy, a similar conformational change could occur upon thiosulfonate formation in sulfonucleotide reductases that would facilitate the recognition and subsequent reduction of this intermediate by thioredoxin*.*

Another related protein modification is the oxidation of cysteine to sulfenic acid (R-S-OH) [37]. This oxidation is reversible and can be reduced by thioredoxin and glutathione. Cysteines can also be hyperoxidized to sulfinic (R-SO_2_H) and sulfonic acids (R-SO_3_H) [37,38]. This process is thought to occur by reaction of susceptible cysteine residues with oxidizing metabolic byproducts, such as hydrogen peroxide. A family of enzymes known as the peroxiredoxins is reversibly inactivated by hyperoxidation of a cysteine residue to the corresponding sulfinic acid in vivo [39,40]. A protein termed sulfiredoxin was later identified and can reduce this sulfinic acid modification back to cysteine [41]. Finally, in humans sulfite is thought to be detoxified by conversion to S-sulfocysteine (Cys-S-SO_3_^−^) [42]. From these few examples, it is apparent that the thiol is subject to unique biological chemistry and is often the site of a diverse array of modifications that include the covalent intermediate we observed in APS reductase.

### The Role of the Iron-Sulfur Center in APS Reductase

All sulfonucleotide reductases analyzed in this study utilized the same overall two-step strategy to catalyze sulfonucleotide reduction. Nevertheless, there are clear differences between APS and PAPS reductases. Specifically, these two classes of enzymes differ in the presence of the iron-sulfur center; APS reductases contain a 4Fe-4S cluster that is notably absent from PAPS reductases. The logical question that emerges from this difference is: What role does the iron-sulfur cluster play in APS reductase?

In this study, we observed ions of *M. tuberculosis* APS reductase that have a measured mass in excellent agreement with the theoretical mass expected for the apoenzyme and the 2Fe-2S intermediate. In support of this assignment, it has recently been demonstrated that exposure to oxygen generates the apo and 2Fe-2S cluster forms of *B. subtilis* sulfonucleotide reductase [19]. In contrast to *M. tuberculosis* APS reductase holoenzyme, these ions do not undergo any mass shift upon addition of substrate. Thus, forms of APS reductase that appear to lack a mature cofactor did not form a stable association with substrate (in contrast to that observed with the Cys249Ser mutant [Figure 8]) or catalyze the formation of the enzyme-thiosulfonate intermediate. These observations are also consistent with previous studies that have correlated the loss of iron with loss of APS reductase activity [18,19]. One explanation for these data is that the loss of cofactor could result in gross active site structural perturbation with concomitant disruption of substrate binding interactions. However, an alternative possibility is that a direct interaction between the iron-sulfur cluster and APS is required for binding. An intriguing corollary of this model could be that PAPS reductases compensate for the lack of this cofactor through additional binding energy gained from the 3′-phosphate moiety of PAPS. In support of this second proposal, all APS reductases reported on thus far exhibit a decrease in activity over time that correlates with decomposition of the iron-sulfur cluster [12,17–19,22]. In one such study, the addition of AMP to *L. minor* APS reductase storage buffer prevented this loss of enzyme activity [17]. A plausible explanation for the additional stability is that the terminal phosphate of AMP interacts with the cluster, preventing oxidation and stabilizing enzyme activity. By analogy, the sulfate moiety of APS could also interact with the cluster.

Inspection of the motif that serves as a marker for the iron-sulfur cluster in APS reductases reveals the presence of an unusual cysteine pair—Cys140 and Cys141 (using *M. tuberculosis* APS reductase numbering). This Cys-Cys tandem motif is atypical within the family of iron-sulfur cluster-containing proteins and has therefore spurred debate as to the number of cysteines that ligate the 4Fe-4S cluster. Mössbauer spectroscopy has been used to probe the cluster in *L. minor* and *P. aeruginosa* APS reductases [12,18]. In these experiments, only three of the four iron sites exhibited similar Mössbauer parameters. Therefore, the authors concluded that the APS reductase 4Fe-4S cluster is ligated by three cysteines. While there is no doubt that the iron-sulfur cluster in APS reductase exhibits unusual spectroscopic properties, alternative interpretations of these data with respect to cluster coordination are possible. The cysteine-labeling studies carried out in this study serve as an independent test of the three-cysteine theory. If only three cysteines participate in iron-sulfur coordination, the simplest expectation would be that two covalent modifications could occur—on Cys141 and Cys249. However, we found that *M. tuberculosis* Cys59Ser and wild-type *P. aeruginosa* APS reductases incorporated only one cysteine modification, specifically at Cys249. These cysteine-labeling data would be consistent with a role for Cys141 in iron-sulfur cluster ligation.

Kim et al. [22] have recently reported on the reaction of *P. aeruginosa* APS reductase with dithio-1,4-nitrobenzoic acid (DTNB). In this experiment, reduction of DTNB gave rise to a spectroscopic signal that was proposed to represent the presence of two free thiols in *P. aeruginosa* APS reductase. As noted above, the data in the present study indicate that *P. aeruginosa* APS reductase has only one free thiol—Cys249. The experiments reported in this work have probed for free thiols using the alkylating reagents VP and iodoacetamide. After the labeling reaction, covalent modifications were quantified by mass spectroscopy. In addition, the identity of labeled cysteines was unambiguously established using site-directed mutagenesis. Unfortunately, quantitation of free thiols by measuring the release of 5-thio-2-nitrobenzoate (TNB^−^) from DTNB by monitoring the absorbance at 412 nm allows for determination only of total thiol content in a sample. In the absence of additional experiments to confirm that a protein cysteine residue has been labeled, and that this label can be eliminated via site-directed mutagenesis, it is not possible to establish the basis of the TNB^−^ signal. For example, iron-sulfur cluster oxidation occurs normally under aerobic conditions and produces free sulfide. Since every mole of APS reductase protein is normally associated with 4 moles of sulfide, decomposition of the cluster can result in significant concentrations of sulfide in the sample [25]. Free sulfide is very reactive, and if present during a DTNB assay, will generate TNB^−^ signal that is not attributable to free protein cysteine thiol content [43]. Furthermore, the study by Kim et al. [22] purified *P. aeruginosa* APS reductase only via affinity chromatography. In the current work, enzymes were purified in an additional step using size-exclusion chromatography. It is also possible, then, that differences in protein preparation could contribute to the observed discrepancy. Interestingly, our analysis indicated that *P. aeruginosa* APS reductase was a homotetramer (Figure S1A).

Additional data obtained from site-directed mutagenesis of Cys140 and Cys141 also support a role for these cysteines in cluster coordination [18,22]. Mutation of Cys140 to serine in *P. aeruginosa* APS reductase results in an enzyme with little detectable iron incorporation. The same mutation to Cys141 results in a less dramatic phenotype, with a 20% decrease in iron and a 35% decrease in sulfide content assayed soon after purification. However, the long-term stability of the iron-sulfur cluster is substantially decreased in the context of the Cys141 mutant as compared to the wild-type enzyme. One explanation for the enhanced short-term stability of the iron-sulfur cluster in *P. aeruginosa* Cys141Ser mutant could involve the homotetrameric nature of this enzyme. Such a higher order assembly could provide stabilizing protein-protein contacts for the iron-sulfur cluster. This hypothesis is supported by the finding that cluster loss in the *P. aeruginosa* Cys141Ser mutant was more rapid at lower protein concentrations [22].

**Figure 13:**
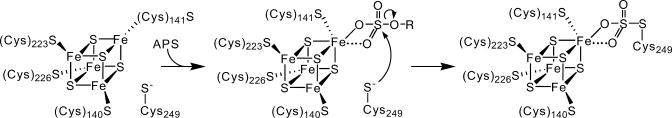
Proposed Role of the Iron-Sulfur Cluster in APS Reduction

Figure 13 illustrates a model that can account both for the unusual properties of the cluster and the role we propose for it in substrate binding. While the Cys-Cys motif is uncommon, a recent computational study has suggested that it is theoretically possible for each of these cysteines to ligate different iron atoms within the same cluster [44]. In addition, the importance of each of the four cysteines is strongly suggested by the cysteine-labeling and site-directed mutagenesis experiments detailed above. Therefore, we propose that in the absence of substrate, each cysteine would participate in cluster coordination. However, the interaction with Cys141 would be unique because of the geometrical constraints imposed by the neighboring cysteine and its own interaction with the cluster. When APS binds the reductase, the oxygens on the sulfate moiety could displace Cys141. In our cysteine-labeling experiments, the addition of substrate blocks labeling by VP; no additional labels are obtained. Thus, for the displacement model to hold true, reaction of VP with the liberated Cys141 would have to be prohibited, for example by the acquisition of a new binding interaction. Alternatively, Cys141 could remain bound, and the additional binding of APS would augment the coordination sphere of the metal. In either scenario, once the substrate is bound, we propose that the unique iron in the cluster acts as a Lewis acid by activating the substrate for subsequent nucleophilic attack and by stabilizing the developing negative charge. A similar role for iron-sulfur cluster participation in substrate binding and activation has been demonstrated for aconitase [45]. This enzyme contains an iron-sulfur cluster with a unique iron site bound by three cysteine ligands and a water molecule. During the conversion of citrate to isocitrate, the terminal oxygen atoms of the substrate interact with this unique iron. However, in our model for APS reductase we are proposing a “substrate-activated” aconitase-like arrangement in which the fourth iron interacts with Cys141; APS binding to the cluster would result in the displacement or shift of this cysteine. An important requirement for iron-sulfur cluster involvement in substrate binding and/or catalysis is that the cluster resides near the active site. Unfortunately, no structural data on assimilatory APS reductases are currently available. However, two studies have reported structural modeling via sequence homology using the crystal structure of *E. coli* PAPS reductase for *P. aeruginosa* and *B. subtilis* APS reductase [19,22]. These models position the cysteines implicated in cluster ligation within the vicinity of the enzyme active site. Thus, the location of the cluster would be consistent with our proposed catalytic function. Nevertheless, additional biochemical investigation together with high-resolution structural information on APS reductase will be required to probe the model presented in Figure 13 in further molecular detail.

### Summary

The evolutionary relatedness within the family of sulfonucleotide reductases has been strongly suggested by sequence homology. Nevertheless, issues of differing substrate specificity, the presence or absence of an iron-sulfur cofactor and the continued conflict in the literature with respect to mechanism had obscured this vision. Hence, the general mechanism of sulfonucleotide reduction established in this work revalidates the evolutionarily relatedness of these enzymes and will help inform future studies to address the experimental challenges outlined above, such as structural characterization, identification of the determinants that govern substrate specificity, and the catalytic involvement of the iron-sulfur cluster. Finally, given that discrete snapshots in the evolution of sulfonucleotide reduction have been biologically retained—APS reductases containing an iron-sulfur cofactor *(M. tuberculosis),* the bispecific APS and PAPS reductase containing an iron-sulfur cofactor *(B. subtilis),* and PAPS reductases without an iron-sulfur cluster cofactor *(E. coli)*—we are presented with an exciting opportunity to probe more general questions regarding the evolution of catalytic diversity in the work to come.

## Materials and Methods

### Materials

Nonradioactive APS was purchased from Biolog Life Sciences Institute, (Bremen, Germany). [^35^S]SO_4_^2−^ (specific activity1,491 Ci/mmol) was obtained from MP Biochemicals (Irvine, California, United States). Molecular biology grade DTT was from Invitrogen (Carlsbad, California, United States). Nonradioactive PAPS, TCEP, and *E. coli* thioredoxin protein were all purchased from EMD Biosciences (San Diego, California, United States). VP, bis-tris propane, methionine, and iodoacetamide were all purchased from Sigma-Aldrich (St. Louis, Missouri, United States). Depending upon availability, PEI-cellulose thin-layer chromatography (TLC) plates (20 cm × 20 cm) were purchased from J. T. Baker (Phillipsburg, New Jersey, United States) or EMD Biosciences. Pfu and DpnI polymerase were from Stratgene (La Jolla, California, United States). Restriction enzymes and T4 DNA ligase were from New England Biolabs (Beverly, Massachusetts, United States). All other chemicals were purchased from J. T. Baker and were of the highest purity available (≥ 95%). DNA oligonucleotides were purchased from Qiagen (Valencia, California, United States).

### Preparation of the sulfonucleotide reductase expression vectors

Table S1 lists the oligonucleotides used in this study. The gene encoding the *M. tuberculosis* APS reductase was amplified from H37Rv *M. tuberculosis* genomic DNA and cloned in a protein expression vector as described [3]. The gene encoding *M. smegmatis* APS reductase was amplified from *M. smegmatis* genomic DNA. The gene encoding *P. aeruginosa* APS reductase was amplified from *P. aeruginosa* genomic DNA ATCC 47085D ( ATCC, Manassas, Virginia, United States). The gene encoding *E. coli* PAPS reductase was amplified from *E. coli* genomic DNA ATCC 700926 ( ATCC). PCR reactions contained 0.25 μM of each primer, 10–100 ng of genomic DNA template, and 2.5 units of Pfu DNA polymerase in reaction buffer supplied by the manufacturer. PCR was performed in a PTC-200 thermocycler (Bio-Rad Laboratories, South San Francisco, California, United States) using the following program: 2 min at 95 °C; 30 cycles of 30 s at 95 °C, 1 min at 55 °C, and 1 min at 68 °C; and a 5-min extension at 68 °C. In some cases, the annealing temperature had to be adjusted in order to achieve the desired product. PCR reactions were screened for the expected DNA fragment via agarose gel electrophoresis. Subsequently, each PCR fragment was ligated into a Zero Blunt Topo cloning vector (Invitrogen) and subsequently digested by NdeI and XhoI (pET24b) or NdeI and BamHI (pET14b). Alternatively, the PCR fragment was directly digested without additional cloning steps. The purified gene fragment was then ligated into a NdeI- and XhoI-digested, CIP-treated pET24b or a NdeI- and BamHI-digested CIP-treated pET14b vector using T4 DNA ligase. The ligation reaction was transformed into chemically competent XL-1 Blue cells (Stratagene). After growth on LB plates containing 50 mg/ml kanamycin (pET24b) or 100 mg/ml ampicillin (pET14b), colonies were selected and cultures were grown up overnight. Plasmid DNA minipreps were screened for the presence of the gene by PCR. DNA sequencing using forward T7 and reverse T7 primers was performed to confirm the identity of the desired gene product.

### Site-directed mutagenesis

Site-specific mutations were made using Quik Change PCR mutagenesis kit (Stratagene) using the appropriate plasmid template according to the manufacturer's specifications. The DNA oligonucleotides used in these reactions are listed in Table S1. Successful incorporation of the desired mutation was confirmed by sequencing and once verified, the plasmid was transformed into BL21(DE3) (Novagen, San Diego, California, United States) for protein expression as described above.

### Expression and purification of sulfonucleotide reductases

Proteins were expressed by transforming a reductase-containing plasmid into BL21(DE3) cells (Novagen) and grown on LB-agarose containing 50 mg/ml kanamycin. An isolated colony was grown in 5 ml of LB broth containing 50 mg/ml kanamycin. The culture was grown at 37 °C overnight. This culture was used to inoculate 1 l of LB broth containing 50 mg/ml kanamycin. The culture was grown with shaking (250 rpm) at 37 °C to an OD of 0.6, and IPTG was added to a final concentration of 0.4 mM. The shaker flasks were then shifted to18 °C and grown 12–16 h. Subsequently, 1 l of cells were collected by centrifugation and resuspended in 30 mls lysis buffer (20 mM sodium phosphate [pH 7.4], 0.5 M NaCl, 10 mM imidazole, and 1 mM methionine) together with an EDTA-free protease inhibitor tablet (Roche, Indianapolis, Indiana, United States) before disruption by sonication on ice. After sonication, DNase and RNase (Sigma) were added to the lysate at 10and 5 μg/ml, respectively, and stirred for 10 min on ice. The cell lysate was cleared by centrifugation and the supernatant was applied to a 1 or 5 ml HiTrap Chelating column (Amersham, Piscataway, New Jersey, United States). The column was washed with ten column volumes in 20 mM phosphate (pH 7.4), 0.5 M NaCl, and 50 mM imidazole, and was eluted with 20 mM phosphate (pH 7.4), 0.5 M NaCl, and 250 mM imidazole. Fractions containing the desired protein were pooled and concentrated using Amicon 10,000 Da molecular weight cutoff centrifugal filters (Millipore, Billerica, Massachusetts, United States) prior to injection onto a 10/30 Superdex 200 or a 16/60 Superdex 200 prep grade gel filtration column. The standard gel filtration buffer was 50 mM Tris-HCl (pH 8.0), 10% glycerol, and 5 mM DTT with ionic strength adjusted to 150 mM with NaCl. The inclusion of DTT in the gel filtration buffer for sulfonucleotide reductases purification has been well established [14–16]. DTT was included in the gel filtration buffer to slow down oxidation and decomposition of the iron-sulfur cluster. No change in retention volume during size exclusion chromatography was observed with any of the proteins purified for this study when DTT was omitted from the buffer. Fractions containing sulfonucleotide reductase were pooled, aliquoted into single-use portions, snap-frozen in liquid nitrogen, and stored at −80 °C. Protein concentrations were determined precisely by quantitative amino acid analysis (AAA Service Laboratory, Boring, Oregon, United States). Iron content, typically ≥ 3.5 mol iron/mol protein, of each preparation was determined as previously described [46,47].

Untagged *M. tuberculosis* APS reductase was expressed as reported above. Cells from 1 l of bacterial culture were resuspended in 30 ml of 20 mM bis-tris propane (pH 7.0) and lysed by sonication. The resulting lysate was clarified by centrifugation, and the supernatant was applied to a 5-ml Fast Flow Q anion exchange column (Amersham). The column was washed with ten column volumes of lysis buffer, and proteins were eluted over ten column volumes with a salt gradient of 0–1 M NaCl. Column fractions were assayed for APS reductase activity and for absorbance at 390 nm to detect the iron-sulfur cluster. APS reductase eluted between 250 and 350 mM NaCl. APS reductase-containing fractions were pooled and ammonium sulfate added to a final concentration of 30%. APS reductase activity was retained in the supernatant. Proteins that precipitated at 30% ammonium sulfate were removed by centrifugation. Ammonium sulfate was added to a final concentration of 70% to precipitate APS reductase. The protein pellet was resuspended in gel filtration buffer and was further purified by size exclusion chromatography, as described above.

### Synthesis of [^35^S]APS and [^35^S]PAPS

^35^S-labeled APS and PAPS were prepared by incubating [^35^S]Na_2_SO_4_, ATP, ATP sulfurylase(Sigma), inorganic pyrophosphatase (Sigma), and recombinant APS kinase together as previously described [48,49]. Analysis of the reaction by PEI-cellulose TLC plates developed in 1 M LiCl indicated complete conversion of [^35^S]SO_4_^2−^ to [^35^S]PAPS. The reaction was then terminated by heating for 2 min in a boiling water bath. The precipitate formed was removed by centrifugation. [^35^S]PAPS was further purified by treatment with activated charcoal as previously described [50]. For the synthesis of [^35^S]APS, nuclease P_1_ was incubated with the [^35^S]PAPS reaction supernatant for 30 min at room temperature. Complete conversion of [^35^S]PAPS to [^35^S]APS was verified by TLC and [^35^S]APS further purified as above. Single-use aliquots of [^35^S]APS and [^35^S]PAPS, always containing less than 1.5% contaminating [^35^S]SO_4_^2−^ , were stored at −80 °C. Minimizing the amount of [^35^S]SO_4_^2−^ present in the substrate served only to facilitate product quantification, as sulfate had no observed effect on reaction rates at concentrations measured into the millimolar regime. Furthermore, while APS is susceptible to nonenzymatic hydrolysis of APS to sulfate and AMP, this reaction was undetectable over the time scale of our assays as measured in reactions that contained only APS or APS incubated with the catalytically inactivated *M. tuberculosis* APS reductase Cys249Ser mutant.

### General kinetic methods

Reactions were performed at 30 °C in assay buffer (50 mM sodium phosphate [pH 7.0], adjusted to 100 mM ionic strength with NaCl) unless noted otherwise. Nonradioactive PAPS or APS were doped with a trace amount of [^35^S]APS or [^35^S]PAPS. Different buffers were used at the same pH value to test for buffer-specific effects on reductase activity. No significant buffer effects on enzymatic activity were observed. Reactions were performed at pH 7.0, the pH value of the maximal rate based on the observed pH dependence of *M. tuberculosis* APS reductase (unpublished data). Multiple turnover reactions contained 10 μM thioredoxin and 5 mM DTT and were typically initiated by the addition of APS or PAPS. As shown by the data presented in Figures 10 and 11 and Table 2, DTT was unable to reduce the thiosulfonate-enzyme intermediate. DTT was included in multiple turnover reactions to regenerate the thioredoxin that becomes oxidized as a consequence of APS reduction (Figure 2). While multiple turnover reactions are typically followed using initial rates, with the exception of very slow or undetectable reactions, we followed our reactions to completion (five half-lives or more). For the purposes of these assays, inhibition by AMP product did not have a significant effect upon the reaction rates measured. The reaction progress curve was plotted as a function of time, and the fractional extent of reaction and fit by a single exponential (Kaleidagraph, Synergy Software, Reading, Pennsylvania, United States). Good first-order fits to the data with end points of 90% were obtained (*R* ^2^ ≥ 0.98). The kinetic data presented in each figure were measured in at least three independent experiments to ensure reproducibility of results.

### TLC-based assay for sulfonucleotide reduction

Traditionally, sulfonucleotide reductase activity has been quantified by quenching the [^35^S]SO_3_^2−^ producing reaction with 2 M sulfuric acid, together with a large excess of cold sulfite, to yield [^35^S]-labeled sulfoxide gas [13,28]. The reaction vessel is rapidly placed into a vial containing an organic base, such as octyl amine, to trap the gas. The vial is closed and incubated overnight to maximize gas absorption. Subsequently, the amount of trapped radiolabeled sulfide is quantified by scintillation counting.

While the original assay continues to be in use, a TLC-based method has more recently been developed that avoids the production of radioactive gas and allows analysis of results on a faster time scale [16]. Perhaps most importantly, the fraction of reaction can be directly determined by quantifying both substrate and product spots as visualized by TLC, which helps to minimize quantification of reaction artifacts and improves the accuracy of measured rates [51]. In this method, a 2-min heat-kill step is used to terminate the sulfonucleotide reductase reaction and is followed by analysis on PEI-cellulose TLC plates developed in 1 M LiCl. To facilitate kinetic analysis of sulfonucleotide reductase activity, we have modified this assay slightly based on methods developed by Peluso et al. to measure GTPase activity [52]. Our modification has been designed to take advantage of the fact that sulfonucleotide reductase activity is pH-dependent; no detectable activity was observed below pH 5 for any of the sulfonucleotide reductases used in this study. At specified times, an aliquot was removed from the reaction mixture and quenched with 0.75 M potassium phosphate (pH 3.3). The products and unreacted substrate were then separated by TLC on PEI-cellulose plates (prepared by prewashing for 5 min in 10% NaCl, followed by three 5-min washes in water and then drying) and developed in 1 M LiCl and 0.3 M sodium phosphate (pH 3.8). Control reactions showed that the quench rapidly extinguished all reductase activity. Under aerobic conditions, free sulfite product naturally oxidizes to sulfate. The rate at which this oxidation occurs is enhanced both by low pH and by the presence of metal ions. Thus, termination of the reaction with the low pH quench and subsequent development in the running buffer described above converts the free [^35^S]SO_3_^2−^ product generated in the enzymatic reaction quantitatively into [^35^S]SO_4_^2−^ . The product was then quantified as [^35^S]SO_4_^2−^ . Addition of 1 mM MgSO_4_ to the quench buffer improved the resolution of substrate and product on the PEI-cellulose plates. TLC plates were analyzed using Phosphorimager analysis (Amersham) with Image Quant quantitation software. In side-by-side experiments, reaction rates measured using the low-pH quench were within experimental error (± 15%) of the previously published heat-kill assay. Representative data for this modified TLC assay are presented in Figure S6.

### Measurement of intermediate formation by TLC

For reactions in which multiple turnover was not desired, thioredoxin was omitted from the reaction mixture. Sulfonucleotide reductase enzyme was used at concentrations in the high nanomolar to low micromolar range to detect a single turnover. Under conditions that had quantitatively produced the thiosulfonate intermediate, E-SO_3_^−^, as observed by mass spectrometry and gel labeling, a new signal on the TLC plate was observed as a discrete spot at that remained the origin of the TLC plate (see Figure S5). In these experiments, intermediate formation was followed in two ways. Since radiolabel would be transferred from substrate to enzyme upon intermediate formation, in the first method we quantified the decrease in APS over time. In a second method, we quantified the appearance of the new spot at the TLC origin, which was assumed to be E-SO_3_^−^. For each enzyme concentration, the reaction endpoint was determined at the point where no further substrate was consumed or intermediate was formed. The endpoint of each reaction (quantified either as fraction APS consumed or as fraction intermediate formed) was plotted as the ratio of enzyme to substrate concentration. As expected, the amount of substrate consumed was identical to the amount of new spot generated, and either treatment of the data yielded the same results.

### Analytical ultracentrifugation

Sedimentation equilibrium experiments were conducted using a Beckman XL-I analytical ultracentrifuge (Beckman Instruments, Fullerton, California, United States). Purified *M. tuberculosis* APS reductase protein in gel filtration buffer was analyzed at concentrations ranging from 5 to 30 μM at 4 °C. All concentrations analyzed gave very similar molecular weights. In independent experiments, samples were centrifuged at speeds ranging from 20,000 to 30,000 rpm, and the absorbance versus radial distribution was determined between 380 and 400 nm, the wavelength region in which the iron-sulfur cluster cofactor bound to the enzyme absorbs. Absorbance data were obtained at the wavelength indicated and at radial increments of 0.003 cm, each data point being an average of three measurements. Data were acquired at intervals over a period of 18 h, with scans at 3-h intervals to ensure that the system had attained equilibrium. Equilibrium distributions were then acquired at 0.001-cm radial increments, each data point being the average of five measurements. The sedimentation equilibrium data were analyzed using a vbar of 0.729 calculated from the amino acid composition, a calculated solvent density of 1.026, and best fit to a model of a single ideal species using a data analysis program supplied by Beckman.

### 4-vinylpyridine and iodoacetamide labeling

*N*-methylmorpholine at final concentration of 10 mM was added to approximately 1 mg/ml protein concentration in gel filtration buffer. Under reducing conditions, either 2 mM TCEP or 5 mM DTT was used. Results obtained were independent of reducing agent used. VP was then added at 2- to 100-fold excess per mole of cysteine residues. The cysteine-labeling reaction proceeded in the dark at room temperature for 60 min and was terminated by snap freezing in liquid nitrogen. For the reactions with substrate, sulfonucleotide was added at a 2- to 5-fold excess concentration per protein monomer prior to the addition of VP. Reactions with iodoacetamide were performed as above, except that iodoacetamide was used at 25 mM final concentration. Labeled samples were buffer-exchanged into NH_4_OAc as described below to remove small molecule reagents, and then diluted with 80:20 acetonitrile:water containing 1% formic acid for mass analysis.

### Buffer exchange and sample preparation for mass spectrometry

Aliquots (100 μl) of purified sulfonucleotide reductase or thioredoxin were buffer exchanged into 50 mM NH_4_OAc (pH 7.5), using Amicon 10,000 Da molecular weight cutoff centrifugal filters with the temperature of the centrifuge set at 4 °C. The buffer was exchanged three times, and the final protein concentration was determined using Bradford assay. Stock solutions of APS, PAPS, and DTT were prepared in 50 mM NH_4_OAc as well. To generate reduced thioredoxin, a ten-fold molar excess of DTT was incubated with the protein for 15 min on ice. Mass analysis was performed to confirm the expected +2 Da shift, relative to oxidized thioredoxin. For enzyme-substrate incubation experiments with *M. tuberculosis* APS reductase, appropriate volumes of each component were mixed in the NH_4_OAc buffer and the mixtures were chilled on ice for at least 15 min before being introduced into the mass spectrometer. For the trypsin digest of APS reductase, 15 μM APS reductase was incubated in 50 mM NH_4_OAc (pH 7.5) with and without 10 molar excess APS at room temperature for 1 h with 0.4% (w/w) sequence grade trypsin. Some mixtures were further diluted with 80:20 acetonitrile:water containing 1% formic acid to detect possible covalent modifications (denatured mass analysis). For the experiments designed to observe intermediate formation and release with *E. coli* PAPS reductase and *P. aeruginosa* APS reductase, incubations were carried out for 15 min at the following concentrations: 10 μM enzyme, 100 μM APS or PAPS, 5 μM thioredoxin, and 50 μM DTT. After incubation, samples were dissolved in 80:20 acetonitrile:water containing 1% formic acid for mass analysis.

### Mass spectrometry

Mass spectrometry data for *M. tuberculosis* APS reductase were acquired on a Bruker FT-ICR MS (Bruker, Billerica, Massachusetts, United States) equipped with an actively shielded 7 tesla superconducting magnet. Solutions were infused at a rate of 2 μl/min into an Apollo electrospray source (Bruker) operated in the positive mode. To analyze the enzyme and its reaction mixture in the native state, the syringe pump and spray chamber were wrapped with ice bags to prevent the protein sample from precipitating out of solution. The N_2_ nebulizing and drying gas pressure was maintained at 50 and 25 psi, respectively. The bias on the glass capillary was kept at 4,600 V, and 140 °C drying gas was used to assist in the desolvation process. Further desolvation was achieved by collisions of the ions with neutral buffer gas at the nozzle-skimmer region using a ∼180-V capillary exit voltage. A throttle valve was installed at the nozzle-skimmer region to adjust the pressure to approximately 1 × 10^−6^ mbar. Ions were externally accumulated in a radio frequency-only hexapole for 1 s before transfer into the ICR cell. Excessive kinetic energy was removed by colliding the ions with Argon pulsed into the cell to a pressure of approximately 1 × 10^−8^ mbar. Usually for one transient, two loops of Argon pulse were applied with a series of pump downs applied to lower the pressure in the cell to approximately 1 × 10^−10^ mbar before ion detection. All ions were collected using gated trapping and detected using chirp excitation and broad band data acquisition using an average of 16–64 time domain transients containing 32 K or 1 M data points. The original time domain free induction decay spectra were zero filled, Gaussian-multiplied and Fourier transformed. All the data were acquired and processed using Bruker Xmass version 6.0.0 software. The parameters of the ESI source, ion optics and cell were tuned for the best signal-to-noise ratio and were kept the same for systematic experiments.

Mass spectrometry data for *E. coli* PAPS reductase and *P. aeruginosa* APS reductase were acquired on a Q-TOF micro mass spectrometer (Waters, Milford, Massachusetts, United States) with electrospray source operated in positive mode. The spray probe used was silicon capillary, which was drawn down to a fine taper above a flame and cut manually to give the required diameter and flow. Samples were infused at a flow rate of 3 μl/min. The ESI source voltages were as follows: 3,200 V capillary, 35 V sample cone, and 1.5 V extraction cone. Source temperature was kept at 120 °C and analyzer pressure read-back was 4.5 × 10^−5^ mbar. A denatured myoglobin solution was used as the calibrant solution. The acquisition range was 500–2,500 *m/z* with an acquisition step of 1.2 s. All spectra were processed using MassLynx software (Waters).

## Supporting Information

Figure S1 Gel Filtration Profile of Wild-Type and Mutant *M. tuberculosis* APS Reductase, *E. coli* PAPS Reductase, and *P. aeruginosa* APS Reductase (A) Superdex 200 protein profile of *M. tuberculosis* APS reductase (filled circles), *E. coli* PAPS reductase (filled squares) and *P. aeruginosa* APS reductase (filled diamonds) followed by absorbance at 280 nm. Inset shows the calibration of the Superdex 200 column with known protein standards (blue dextran 2,000,000 Da, thyroglobulin 670,000 Da, bovine IgG 158,000 Da, human IgG 150,000 Da, bovine serum albumin 67,000 Da, ovalbumin 44,000 Da, chymotrypsin 25,000 Da, myoglobin 17,000 Da, and ribonuclease A 13,700 Da). Based on elution volume, the molecular weight (calculated from the standard curve of known protein standards) of *M. tuberculosis* APS reductase is 34,033 Da, *E. coli* PAPS reductase is 69,085 Da, and *P. aeruginosa* APS reductase is 128,078 Da. For each sulfonucleotide reductase the gel filtration profile of the catalytic cysteine to serine mutant has also been plotted (*M. tuberculosis,* open circles; *E. coli,* open squares; and *P. aeruginosa,* open diamonds).
(B) ESI-mass spectrometry spectrum of 10 μM APS reductase in 50 mM NH_4_OAc. Three charge states are observed, 9+, 10+ and 11+. The calculated mass is 28,706.00 Da using the deconvolution function on the Bruker Xmass software.
(2.3 MB EPS).

Figure S2 Gel Filtration and Activity Profile of Untagged and His-Tagged *M. tuberculosis* APS Reductase (A) Superdex 200 profile of untagged *M. tuberculosis* APS reductase, purified in prior steps by anion exchange and ammonium sulfate fractionation, was followed by analysis of absorbance at 390 nm to detect the iron-sulfur cluster (solid line). The predicted molecular weight of the major peak is 34,334 Da, close to the expected value of 27,638 Da. Assay of untagged *M. tuberculosis* APS reductase activity across the gel filtration column (dashed line) as described in Materials and Methods.
(B) Activity of His-tagged *M. tuberculosis* APS reductase plotted in conjunction with absorption at 390 nm.
(708 KB EPS).

Figure S3 Gel Labeling of Wild-Type, Cys59Ser, and Cys249Ser *M. tuberculosis* APS Reductase In this experiment, 5 μM wild type (lanes 1–3), Cys249Ser (lanes 4–6), and Cys59Ser (lanes 7–9) *M. tuberculosis* APS reductase was incubated at room temperature in 50 mM bis-tris propane (pH 7.0), 100 mM NaCl with [^35^S]APS only (lanes 1, 4, and 7); with 200 μM APS for 5 min prior to the addition of [^35^S]APS (lanes 2, 5, and 8); or with [^35^S]APS for 5 min followed by the addition of 10 μM thioredoxin (lanes 3, 6, and 9). SDS-PAGE load dye (without reductant) was added to terminate the reaction. The samples were heated at 60 °C for 3 min and size-fractionated by 12% nonreducing SDS-PAGE. The incorporation of radioactivity was analyzed with a Phosphorimager.
(2.1 MB TIF).

Figure S4 Gel Labeling of Wild-Type and Mutant *E. coli* and *P. aeruginosa* Sulfonucleotide Reductases (A) Gel labeling of wild type (lanes 1–3) and Cys239Ser (lanes 4–6) *E. coli* PAPS reductase was carried out as described in the legend to Figure S3, except that PAPS was used as the substrate.
(B) Gel labeling of wild type (lanes 1–3) and Cys256Ser (lanes 4–6) *P. aeruginosa* reductase was carried out as described in the legend to Figure S3.
(1.7 MB TIF).

Figure S5 Intermediate Formation and Sulfite Release by *M. tuberculosis*, *E. coli,* and *P. aeruginosa* Sulfonucleotide Reductases (A) Here, 5 μM *M. tuberculosis* APS reductase (lanes 3–6) or *E. coli* PAPS reductase (lanes 9–12) was incubated at room temperature in 50 mM bis-tris propane (pH 7.0), 100 mM NaCl with [^35^S]APS or [^35^S]PAPS in the presence (lanes 5, 6, 11, and 12) or absence (lanes 3, 4, 9, and 10) of 10 μM thioredoxin. Lanes 1, 2, 7, and 8 are control reactions in which sulfonucleotide reductase and thioredoxin have been omitted. Each odd lane is a sample of the reaction prior to addition of sulfonucleotide reductase. Each even lane is a sample from the reaction that has been terminated 10 min after the addition of sulfonucleotide reductase. No intermediate release was observed using 10 mM DTT in place of thioredoxin (unpublished data).
(B) Experiment exactly as above except with *P. aeruginosa* APS reductase.
(3.7 MB EPS).

Figure S6 Multiple Turnover TLC Assay for Sulfonucleotide Reduction (A) TLC analysis to follow the extent of the sulfonucleotide reductase reaction, as described in Materials and Methods. A complete reaction progress curve for the reaction of trace [^35^S]APS, 20 μM APS, 10 μM thioredoxin, 5 mM DTT, and 20 nM *M. tuberculosis* APS reductase. The relative amounts of ^35^S in the product and substrate spots were quantified with a Phosphorimager.
(B) The time course of the reaction shown in (A) follows a single-exponential function.
(2 MB TIF).

Table S1 Oligonucleotide Primers Used in This Study (33 KB DOC).

### Accession Numbers

Available GenBank accession numbers for the sulfonucleotide reductase genes discussed in this paper are as follows: *M. tuberculosis* (CAB03733.1), *R.* *meliloti* (AAD55759.1), *P. aeruginosa* (AAG05145.1), *B. cepacia* (AF170343), *L. minor* (CAB65911.1), *A. thaliana* (APR2) (AAC26977.1), *E. coli* (BAB37040.1), *S. flexneri* (AAP18092.1), *S. cerevisiae* (AAA34774.1), *B. subtilis* (CAB13431.1), and *B.* *anthracis* (AAT30539.1).

## Abbreviations

4Fe-4S: four iron-four sulfur cluster
AMP: adenosine 5′-phosphate
APS: adenosine 5′-phosphosulfate
Cys: cysteine
Da: Dalton
DTNB: dithio-1,4-nitrobenzoic acid
DTT: dithiothreitol
E: enzyme
ESI: electrospray ionization
FT-ICR: Fourier transform ion-cyclotron resonance
His: histidine
PAPS: 3′-phosphoadenosine 5′-phosphosulfate
S: substrate
Ser: serine
TCEP: tris-(2-carboxyethyl)phosphine
TLC: thin layer chromatography
TNB^−^: 5-thio-2-nitrobenzoate
VP: 4-vinylpyridine
