# Supplementary material for: A Conserved Mechanism for Sulfonucleotide Reduction
Source: PLoS Biol. 2005 Jul 19;3(8):e250. doi: 10.1371/journal.pbio.0030250 (PMC1175818; doi:10.1371/journal.pbio.0030250)
Supplement: Supplementary file 1 [file pbio.0030250.st001.doc]

**Table S1** Oligonucleotide primers used in this study.

| Primer | Mutation*^a^* | Primer Sequence (5’🡪3’)*^b,c^* |
| --- | --- | --- |
| *E. coli* Start |  | cat**atg**tccaaactcgatc |
| *E. coli* End |  | ggatccttacccttcgtgcaatc |
| *P. aeruginosa* Start |  | cat**atg**ctgccctttgctacc |
| *P. aeruginosa* End |  | ctcgagggccttgctgatcag |
| *M. tuberculosis* | Cys59Ser | gggtggacaacgtccaactacgtagttgc |
| *M. tuberculosis* | Cys249Ser | ggctggccaagaccgaatccgggttgcacgcctc |
| *E. coli* | Cys239Ser | GCTTAAAAAGGGAATCTGGGTTACACGAAGG |
| *P. aeruginosa* | Cys256Ser | CCACAAGGAGTCCGGGCTGCACG |

*^a^* Numbered from the beginning of the sulfonucleotide reductase start codon.

*^b^* Sequence in boldface indicates start codon.

*^c^* A pair of complementary primers was used for each mutant, the reverse complements are not shown. Changes to the sequence are underlined.
